# PKM2 Subcellular Localization Is Involved in Oxaliplatin Resistance Acquisition in HT29 Human Colorectal Cancer Cell Lines

**DOI:** 10.1371/journal.pone.0123830

**Published:** 2015-05-08

**Authors:** Alba Ginés, Sara Bystrup, Vicenç Ruiz de Porras, Cristina Guardia, Eva Musulén, Anna Martínez-Cardús, José Luis Manzano, Laura Layos, Albert Abad, Eva Martínez-Balibrea

**Affiliations:** 1 Translational research in digestive tumours group, Laboratory of Molecular Cancer Biology, Health Sciences Research Institute of the Germans Trias i Pujol Foundation (IGTP), Badalona, Spain; 2 Human Pathology Department, University Hospital Germans Trias i Pujol, Badalona, Spain; 3 Medical Oncology Service, Catalan Institute of Oncology (ICO) University Hospital Germans Trias I Pujol, Badalona, Spain; Sapporo Medical University, JAPAN

## Abstract

Chemoresistance is the main cause of treatment failure in advanced colorectal cancer (CRC). However, molecular mechanisms underlying this phenomenon remain to be elucidated. In a previous work we identified low levels of PKM2 as a putative oxaliplatin-resistance marker in HT29 CRC cell lines and also in patients. In order to assess how PKM2 influences oxaliplatin response in CRC cells, we silenced PKM2 using specific siRNAs in HT29, SW480 and HCT116 cells. MTT test demonstrated that PKM2 silencing induced resistance in HT29 and SW480 cells and sensitivity in HCT116 cells. Same experiments in isogenic HCT116 p53 null cells and double silencing of p53 and PKM2 in HT29 cells failed to show an influence of p53. By using trypan blue stain and FITC-Annexin V/PI tests we detected that PKM2 knockdown was associated with an increase in cell viability but not with a decrease in apoptosis activation in HT29 cells. Fluorescence microscopy revealed PKM2 nuclear translocation in response to oxaliplatin in HCT116 and HT29 cells but not in OXA-resistant HTOXAR3 cells. Finally, by using a qPCR Array we demonstrated that oxaliplatin and PKM2 silencing altered cell death gene expression patterns including those of BMF, which was significantly increased in HT29 cells in response to oxaliplatin, in a dose and time-dependent manner, but not in siPKM2-HT29 and HTOXAR3 cells. BMF gene silencing in HT29 cells lead to a decrease in oxaliplatin-induced cell death. In conclusion, our data report new non-glycolytic roles of PKM2 in response to genotoxic damage and proposes BMF as a possible target gene of PKM2 to be involved in oxaliplatin response and resistance in CRC cells.

## Introduction

Colorectal cancer (CRC) remains one of the most frequent causes of cancer-related death worldwide. The 5-year overall survival rate is less than 10% in advanced stages of the disease and chemotherapy treatment remains essential for these patients. Despite the availability of new target therapies against EGFR or VEGF, combinations of oxaliplatin (OXA) with fluoropyrimidines remain the most commonly used frontline regimens in the metastatic setting [1, 2]. Cytotoxicity of OXA is mainly generated through the formation of platinum-DNA adducts resulting in DNA transcription and replication blockade. Consequently, it activates several signaling pathways leading to DNA damage repair and/or the activation of cell death programs [3] which in turn depends, among other factors, on the mutational status of the tumor suppressor gene p53 [4–6]. However, it is apparent that not all patients benefit from OXA treatment with resistance processes representing the main obstacle of treatment effectiveness. Chemoresistance to platinum agents is a complex and multifactorial process in which several mechanisms such as drug influx/efflux modifications, alterations in DNA damage repair, decrease of cell death activation, autocrine survival signaling or high detoxification activity could take part [7–10]. Unfortunately, most of the studies concerning platinum drugs resistance have focused on cisplatin and the real biological behavior and mechanisms of response to OXA in colorectal cells is mostly unknown.

In the past few years many studies have directed their attention to tumor cell metabolism as a mechanism of cell adaptation to drug sensitivity [11, 12]. In this line, we found in a previous study that isoform M2 of Pyruvate Kinase enzyme (PKM2) is linked to OXA resistance acquisition in an *in vitro* model and we were able to translate our results into a small cohort of metastatic CRC patients who had received OXA/5-FU chemotherapy [8]. Other authors have reported that PKM2 expression and activity is linked to cisplatin resistance in gastric tumor cells [13] and in colorectal cancer cells with acquired resistance to 5-FU treatment [14]. These facts indicate that this enzyme could have an important role in resistance acquisition processes to different chemotherapeutic drugs. Furthermore, it has been shown that some of the PKM2 biological functions depend on the enzyme’s nuclear translocation which is promoted by different post-translational modifications such as tyrosine phosphorylation [15–17], lysine acetylation [18], or sumoylation [19] in response to the factors EGFR [20], IL-3 [21] or Oct-4 [22] respectively. While in the majority of the above mentioned cases PKM2 translocation results in the stimulation of cell proliferation, it has been demonstrated that after other kinds of stimuli like DNA damage or oxidative stress, PKM2 translocates to the nucleus of cells leading to the activation of cell death in a caspases and Bcl-2 independent manner [23].

In the work presented here, we wanted to elucidate the PKM2-related molecular mechanisms responsible for OXA resistance acquisition in an *in vitro* model previously described by us [24]. As it will be shown, modulation of PKM2 expression altered OXA sensitivity not only in this cellular model but also in other human CRC cell lines. We show that PKM2 translocates to the nucleus in response to genotoxic damage caused by OXA in sensitive but not in cell lines with acquired resistance to the drug and it regulates the expression pattern of cell death genes such as BMF, which has been shown to be involved in the activation of apoptotic and non-apoptotic cell death.

## Materials and Methods

Signed informed consent was obtained from each patient, and the Clinical Research Ethical Committee from Hospital Germans Trias I Pujol provided approval for the study.

### Cell lines

HCT116 colon carcinoma cells and its isogenic derivative with a targeted inactivation of p53 were a gift of Dr Vogelstein (Johns Hopkins University School of Medicine). SW480 and HT29 were obtained from the American Type Culture Collection (Manassas, VA). The latter was used as the parental cells of the OXA-resistant subline HTOXAR3, which was obtained as a result of continuous and increased exposure to OXA as described previously [9]. Cell lines were grown as monolayers in DMEM (HT29, SW480 and HTOXAR3; Invitrogen, Life Technologies) or RPMI 1640 (HCT116 and HCT116 p53 null; Invitrogen, Life Technologies) supplemented with 10% heat-inactivated FCS (Reactiva), 400 units/ml of penicillin, 40 μg/ml of gentamycin, and 2 mM of L-glutamine (Sigma) and cultured at 37°C in a humidified atmosphere of 5% CO_2_. Cells were periodically tested for contamination by *Mycoplasma* and were authenticated by short tandem repeat profiling.

### Drugs

OXA was prepared in water (1 mM) as stock solution and stored at -20°C. Further dilutions of the drug were made in culture medium to final concentrations before use.

### siRNA transfections

Cells were seeded at 60% confluence in serum and antibiotic-free OptiMem medium (Invitrogen) in 6, 12 and 96 well plates, depending on the following experiments. PKM2 was transiently silenced by using three different siRNAs targeting PKM2 (seq. NM_002654; NM_182470; NM_182471; Ambion). P53 and BMF inhibition was carried out with pools of 4 different siRNAs (Smartpool On-target plus: *TP53*, #L-003329; *BMF*, #L-004393-00, Dhamacon, GE). As a transfection agent we used Lipofectamine RNAiMAX (Invitrogen) according to the manufacturer’s instructions. A silencer negative transcription control (Cat No. AM4611; Ambion) was introduced in each experiment. Following 24 h of transfection, medium was replaced with full DMEM or RPMI 1640 mediums supplemented with serum and antibiotic. Validation of PKM2, p53 and BMF knockdown was assessed by qPCR and Western blot (WB), only by WB and only by qPCR, respectively. In fine-tune experiments, siGAPDH 3 nM positive control (NM_002046; Ambion) and non-transfected cells (Mock) were introduced to ensure that transfection had minimal effects on gene expression, proliferation and cell viability (S1 Fig).

### Western blot

Cells were homogenized in RIPA plus buffer [Phosphate Buffered Saline (PBS); NP-40 1%; Na deoxycolate 0.5%; SDS 0.1%; EDTA 1 mM; NaF 50 mM; NaVO_3_ 5 mM] containing a cocktail of EDTA-free protease inhibitors (Roche). Protein concentration was determined by the Bradford method by using the BCA Protein Assay Kit (Pierce) and bovine serum albumin as a standard. Twenty micrograms of protein were loaded and subjected to electrophoresis in 10% SDS-PAGE gels (Invitrogen) and transferred onto PVDF membranes (Bio Rad). After 1 h of blocking (LICOR Biosciences) membranes were incubated overnight at 4°C with a rabbit polyclonal anti-PKM2 primary antibody (Cell signaling; 1:1000) or with a mouse monoclonal anti-p53 (Abcam; 1:500). Rabbit monoclonal anti-Actin (1:2000) and mouse monoclonal anti-α-Tubulin antibodies (1:15000) (both from Sigma Aldrich) were used as internal controls. Membranes were incubated with IRDye rabbit and mouse secondary antibodies (1:15000) (LICOR Biosciences) for 45 minutes protected from light. Membranes were scanned by using Odyssey Imaging System and analyzed with Odyssey v2.0 software (LICOR Biosciences).

### qPCR

Gene expression experiments were performed as described in a previous work [24]. Retrotranscription was performed with MMLV reverse transcriptase (Invitrogen) in accordance to the manufacturer’s instructions. Primers and probes for PKM2 (assay no. Hs00762869_s1) and BMF (Hs.00372938_m1) mRNA expression analysis were purchased predesigned from Applied Biosystems. The PCR product size generated with these primers was 62 bp for PKM2 and 59 bp for BMF. Relative gene expression quantification was calculated according to the comparative Ct method as described elsewhere using 18S (Stratagene) or β-Actin (Applied Biosystems) as endogenous controls. In all experiments, only triplicates with a Ct value lower than 0.20 SD were accepted. In addition, for each sample analyzed, a retrotranscriptase minus control was run in the same plate to ensure absence of genomic DNA contamination.

### MTT

The cytotoxicity of OXA was assessed by the 3-(4, 5-dimethylthiazol-2-yl) 2,5-diphenyltetrazolium bromide (MTT) test. Cells were seeded and transfected in 96-well microtiter plates (Nunc) at a density of 1,000 (HT29 and SW480) and 2,000 cells/ well (HCT116). Forty eight hours after siRNA transfection, OXA was added at different concentrations; cell viability was determined 24 h after incubation by the MTT assay (Roche Diagnostics) [25]. Inhibitory concentrations (ICs, ranging from 10% to 90% of cell viability) were determined in each cell line by the median-effect line method. The data reported represent the mean ± SD of a minimum of three independent experiments.

### Trypan Blue stain

Cells were grown in 12 well plates and treated with 15 μM OXA for 24 h. After drug exposure cells were harvested and resuspended in DMEM medium at a concentration 1x10^5^ cells/ml. Cytotoxicity was assessed using trypan blue staining. Ten microliters of 0.05% trypan blue (Invitrogen) was mixed with 10 μl of cell suspension, spread onto a Neubauer chamber and covered with a coverslip. While viable cells exclude the dye and appear translucent, nonviable cells appear blue stained. Viability and mortality of at least 3 replicates of each experimental condition were quantified.

### Annexin V/ Propidium Iodide test

Apoptosis was determined by using FITC Annexin V Apoptosis Detection Kit I (BD Pharmingen) according to the manufacturer’s instructions. A minimum of 10^4^ cells per sample was analyzed by using FACS Canto II flow cytometer (Becton Dickinson Immunocytometry System). At least 3 replicates per experimental condition were analyzed. Positive and negative controls (binding buffer only, propidium iodide (PI) only, FITC-Annexin V only) were used to set up appropriate conditions to compensate detectors and quadrants. OXA-induced cell death after BMF gene silencing was measured by using PI. BMF was silenced using siRNA targeting BMF as described above. After 72 h Oxaliplatin treatment, HT29 cells were harvested with Accutase (Ref. A11105-01, Invitrogen) and resuspended in cold PBS with a PI concentration of 3μM. The PI fluorescence was determined on a FACSCanto II flow cytometer (Becton Dickinson Immunocytometry System). BMF gene silencing was confirmed by qPCR.

### Cell cycle analysis

To assess cell cycle distribution cells were harvested, washed in PBS, fixed in 1 ml 70% ice-cold ethanol and stored at 4°C for at least 30 min. Pellets were resuspended in 0.5 ml of 0.1 M HCl buffer and incubated for 10 minutes at 37°C. Reactions were stopped with 2.5 ml PBS. Cells were incubated in 1 ml of PI staining solution (30 μM (Applichem); RNAse A 200 μg/ml (Sigma)) for at least 30 min at room temperature in the dark. A minimum of 10,000 cells was analyzed for DNA content using a FACS Canto II flow cytometer (Becton Dickinson Immunocytometry System). The proportion of cells in G1, S phase and G2/M was determined using Flowjo Software v9.2.

### Immunofluorescence analysis

PKM2 subcellular localization was detected by immunofluorescence. After 24 h of attachment in cell chamber slides (Millipore), cells were treated with OXA and fixed to coverslips in cold acetone for 10 min at room temperature. Blocking and permeabilization was done with PBS-T/ FBS 10%. Cells were incubated at room temperature with a rabbit polyclonal anti-PKM2 primary antibody (Cell Signaling; 1:100) for 1.5 h and subsequently, with secondary antibody anti-rabbit Alexa-568 (Invitrogen; 1:200). Nuclei were stained with DAPI gold-antifade reagent (Invitrogen). Coverslips were observed with a fluorescence microscope Axiovision Z1 by using Apotome system at 40x immersion oil lens (Carl Zeiss, Heidelberg, Germany). Multiple images were taken at different focus distances by using z-stacking (thickness interval: 0.750–1 μm) to localize PKM2 at different focal depths.

### qPCR array

Quantitative real-time PCR (qRT-PCR) was performed by using Human Cell Death Pathway Finder PCR Array 384 HT (PAHS-212Z, SA Biosciences), a qPCR Array containing 84 cell death-related genes. Briefly, Total RNA was collected from cells using EZNA total RNA Kit I (Omega) and treated with DNAse (Ambion). RNA was quantified with a Nanodrop TM ND-1000 spectrophotometer (Thermo Scientific). RNA integrity and lack of genomic contamination were assessed by running samples in 1% agarose gels. A total of 500 ng RNA was used for reverse transcription with RT^2^ First Strand Kit (SA Biosciences). PCR reactions were performed using the RT^2^ profiler PCR array mentioned before and RT^2^ Real-time SYBR Green PCR master mix (SA Biosciences) on a 7900HT Fast Real-time PCR system (Applied Biosciences) and following manufacturer’s instructions. Relative changes in gene expression were calculated using the 2^^-ΔCt^ (threshold cycle) method. Those housekeeping genes that did not show variability between experimental conditions were selected to be included on the array (RPLP0 and ACTB) to normalize the cDNA amounts. Three independent biological replicates were performed.

### Statistical analysis

Statistical Analysis was carried out using PASW statistics 18 (IBM) except for the dose-response curves analysis, which was carried out with PRISM4 program (Graphpad Software). Statistical differences between IC_50_ were determined by graphic representation of dose-response curves and subsequent non-linear regression analysis and F test. U-Mann Whitney test was used to determine cell cycle distribution differences. Comparisons among different experimental conditions in qPCR Array and BMF expression experiments were carried out through the T-Student test. Differences were considered statistically significant at *P < 0*.*05*.

## Results

### PKM2 gene silencing alters OXA sensitivity of human colorectal cancer cells

**Fig 1:**
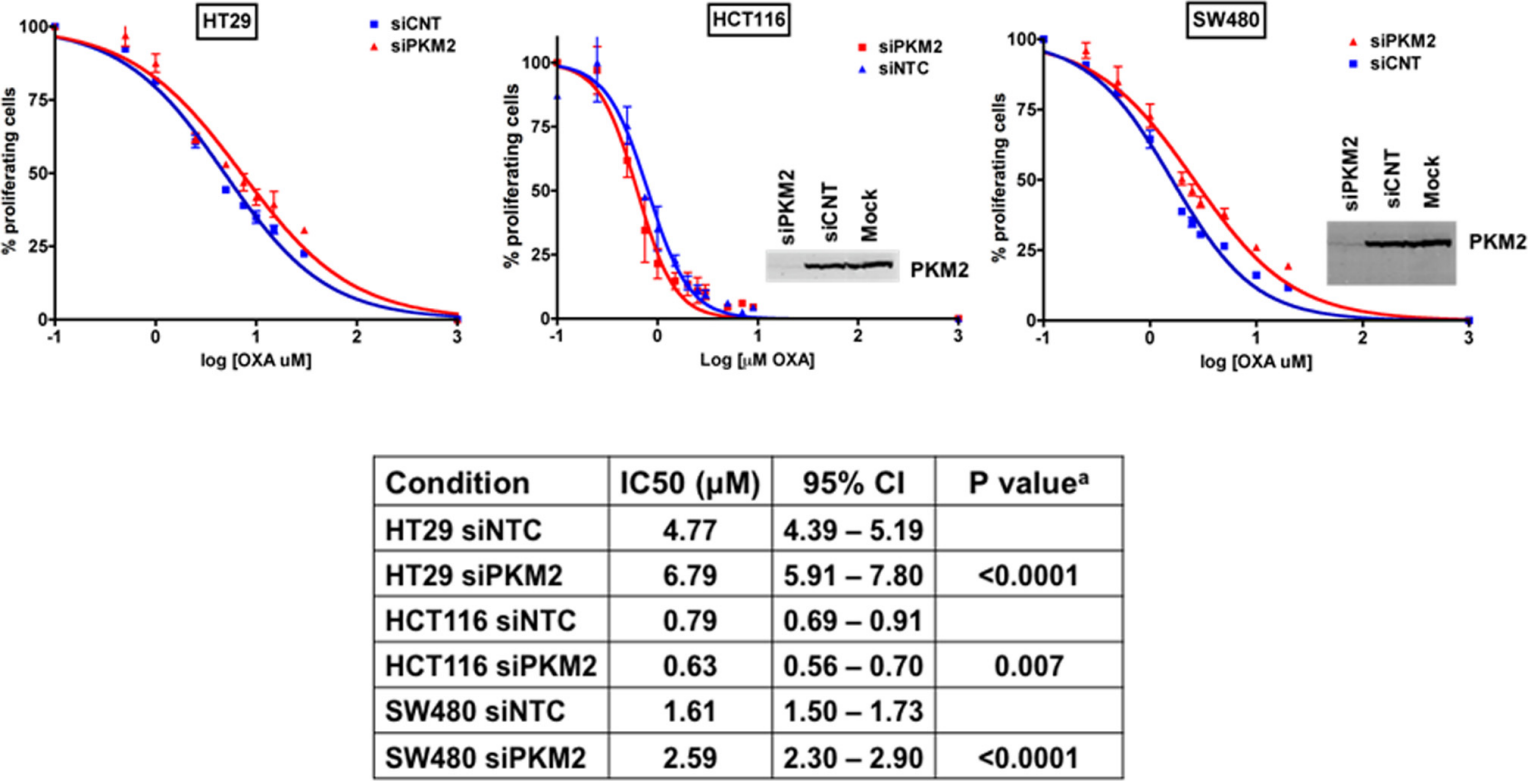
Effect of PKM2 silencing on OXA cytotoxicity in HT29, SW480 and HCT116 cells. Dose-response curves for HT29, SW480 and HCT116 cell lines after PKM2 gene silencing and OXA treatment at 0–140 μM and 0–32 μM for 24 hours. Curves represent the average values from at least three independent experiments. Cell proliferation was measured by MTT assay. Vertical bars in the graphics represent ± SD. Insets show PKM2 inmunoblotting after siRNA-directed inhibition. Specific IC50 values for oxaliplatin in all conditions are displayed in the table. IC50 values for Mock conditions (without transfection) were very similar to those of siNTC and are not shown. *p-values are result of comparison to the siNTC condition.

**Fig 2:**
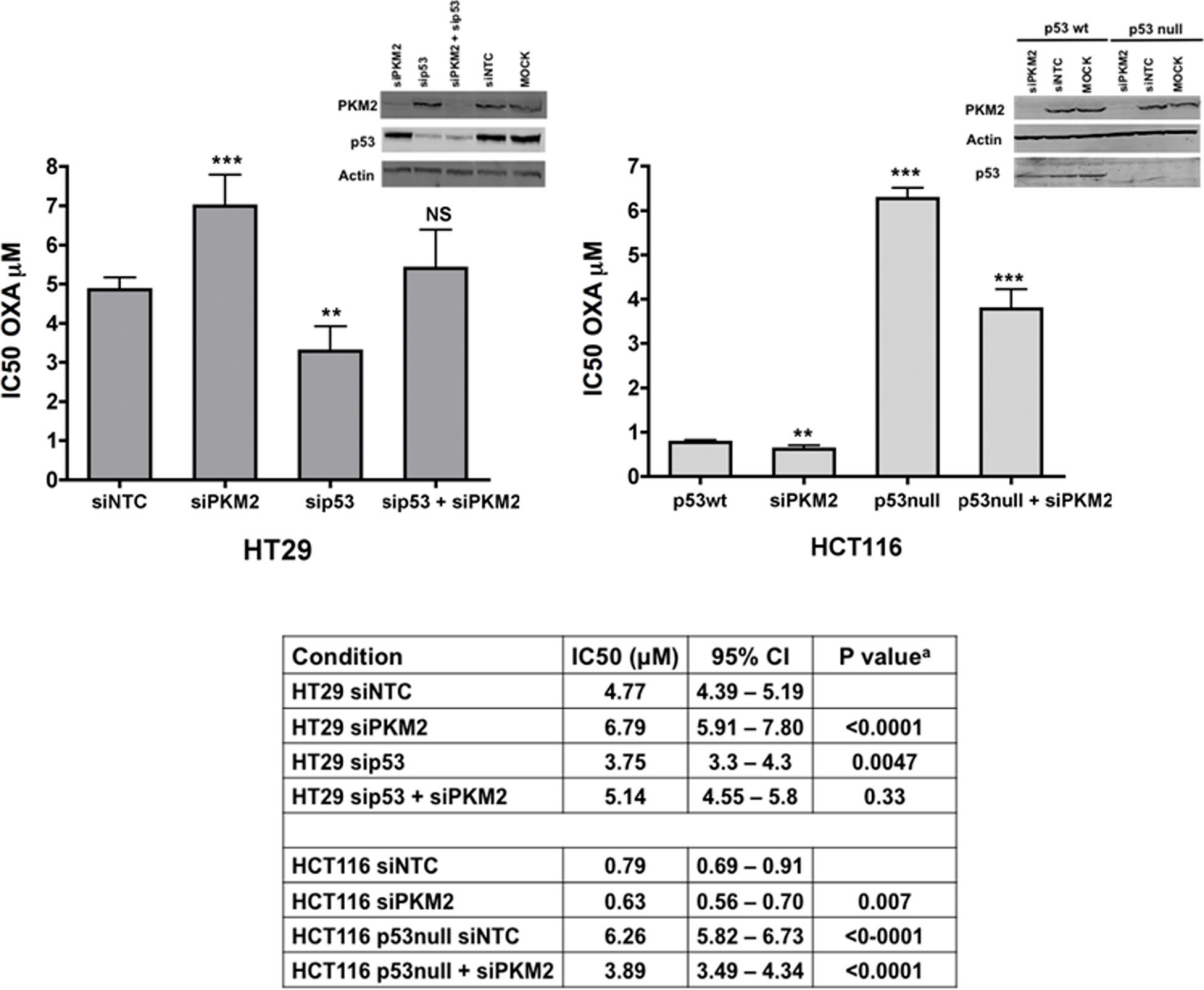
Effect of PKM2 silencing according to p53 in HT29 and HCT116 cell lines. Bars represent the IC50 ± SD (average values from at least three independent experiments) for oxaliplatin for each condition. Insets show PKM2 and p53 inmunoblotting after PKM2 gene silencing. Specific IC50 values for oxaliplatin in all conditions are displayed in the table. IC50 values for Mock conditions (without transfection) were very similar to those of siNTC and are not shown. *p-values are result of comparison to the siNTC condition. * *P- value* < 0.05. ** *P-value <* 0.01; *** *P-value <* 0.001

In a previous work we found lower levels of PKM2 protein and mRNA expression in CRC cells with acquired resistance to OXA (HTOXAR3) and in tumors from OXA non-responder patients, especially in those with mutated p53 [8]. In order to assess the effect of down-regulating PKM2 on OXA response in parental cells, we specifically inhibited PKM2 gene expression by using siRNA oligonucleotides in HT29 cells. Sensitivity to OXA in control (siNTC) and PKM2 knockdown cells (siPKM2) was compared by MTT assay. Forty-eight hours after gene silencing, cells were seeded in 96-well plates and treated for 24 h with OXA doses ranging 0–30 μM. PKM2 knockdown efficiency was higher than 95% at both mRNA and protein levels and lasted for 96 h or more (S1 Fig). As it can be seen in Fig 1, in HT29 cells, PKM2 silencing led to more than 40% increase in OXA resistance as compared to siNTC cells (fold = 1.42; p<0.0001) confirming the influence of PKM2 down-regulation in oxaliplatin resistance in these cells. These results were validated in SW480 cells (fold = 1.61 p<0.0001) but not in HCT116 where PKM2 knockdown was associated with higher sensitivity to OXA (fold = 0.80; p = 0.007) (Fig 1). We hypothesized that PKM2 influenced the tumor cells’ response to OXA depending on additional factor/s. One one possibility could be the mutational status of p53 since all these cell lines have abnormal EGFR signaling pathway (HT29 harbors mutations in BRAF and PI3K, HCT116 in KRAS and PI3K and SW480 in KRAS) but present different p53 mutational status (HT29 and SW480 are p53 mutated and HCT116 are p53 wt). In order to demonstrate this hypothesis, we used an HCT116 isogenic derivative with targeted inactivation of p53 (HCT116 p53 -/-)[26]. p53 null cells were highly resistant to oxaliplatin as previously reported [27] but the inhibition of PKM2 gene expression again led to a decrease in oxaliplatin resistance (Fig 2). These results indicated a possible effect not depending on wt p53 but on a gain-of-function (GOF) mutation of p53. According to this, we expected a change in oxaliplatin sensitivity in HT29-siPKM2 cells after silencing p53 gene expression. As it is shown in Fig 2, p53 gene knock down in HT29 cells (siNTC) led to a decreased resistance to oxaliplatin, but under these conditions the lack of PKM2 expression also increased resistance in these cells. Taken together these results suggest that the role of PKM2 in oxaliplatin sensitivity is cell line-dependent and that other factors, different from mutated p53 *per se*, but probably associated with a p53-mutated carcinogenic context, could be influencing the different behavior observed in these cell lines after PKM2 gene silencing. Further experiments are warranted in order to demonstrate this point.

### PKM2 gene silencing in HT29 cells is associated with an increase in cell viability but not with a decrease in apoptosis after OXA exposure

**Fig 3:**
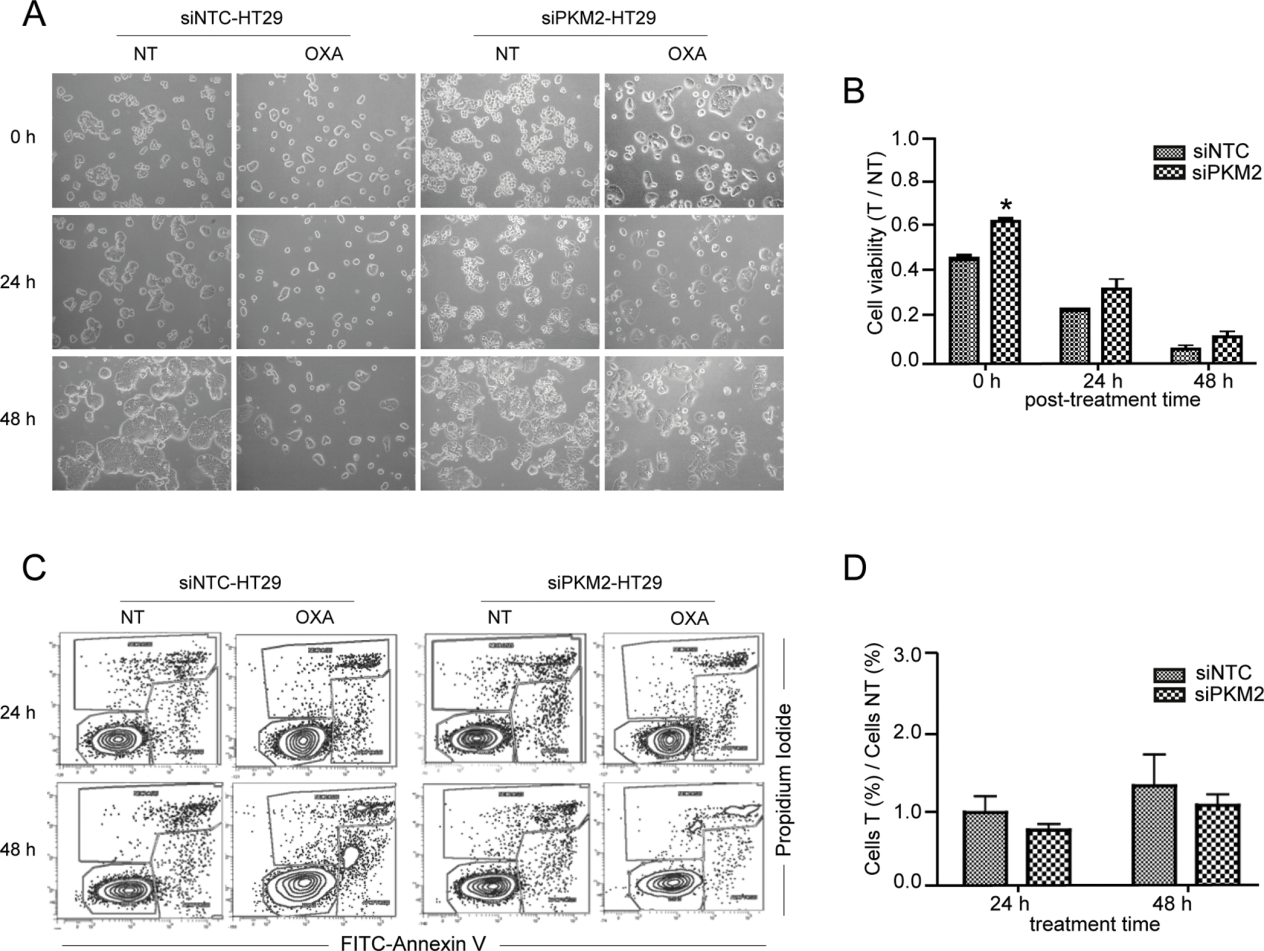
PKM2 silencing alters OXA response in HT29 cells but not apoptosis activation. After siRNA transfection cells were treated with 15 μM OXA for 24 h period, observed by optical microscopy at 0, 24 and 48 h after the end of drug exposure (0 h refers to cells treated for 24h; 24 h refers to cells treated for 24 h and left to recover for additional 24 h) (A) and quantified by trypan blue staining (B). Apoptosis activation after 24 and 48 hours of 10 μM OXA exposure was determined by FITC-Annexin V/ PI double staining (C) and measured as a ratio between percentages of apoptotic treated (T) and non-treated (NT) cells (D). Vertical bars in the graphics represent ± SD. *P-value < 0.05. NT: non-treated cells. Optical microscopy: objective 10x magnification.

Following our main objective, we wanted to know whether the effect of PKM2 gene silencing on oxaliplatin resistance in our *in vitro* model, was due to an increase in cell viability, to a decrease in apoptosis or both. PKM2 was silenced in HT29 cells before treating them with 15 μM OXA for 24 hours. The effects were compared to control cells treated the same way. By using trypan blue staining, viability rates between OXA treated (T) and non-treated (NT) cells were calculated for 0, 24 and 48 h recovery times (after treatment with oxalplatin, cells were left to recover for 0, 24 and 48 h, respectively). Twenty-four hours after treatment (time point 0h) a clear effect of OXA was detected. Cell population of siNTC and siPKM2 cells diminished progressively after 48 h treatment. However, viability was slightly higher in siPKM2 cells compared to siNTC cells at all time-points, which was statistically significant at 0 h (Fig 3A and 3B). This fact indicates that PKM2 is affecting OXA response in these cells. To further investigate downstream mechanisms associated with PKM2-related increase in viability in response to OXA, we analyzed the effect on apoptosis by using the Annexin V/ PI double staining assay (Fig 3C). Cytotoxicity induced by OXA did not markedly alter levels of induced early apoptosis until 48 hours after continuous exposure (Fig 2D). Moreover, we did not find significant differences in apoptosis activation between silenced PKM2 and control cells in presence of OXA but there was a trend that siNTC cells died in a larger proportion than siPKM2 cells. These results reinforce the idea that PKM2 participates in OXA resistance and that apoptosis is not the principal pathway implicated in cell death activated after OXA exposure in this cell line.

### Cell cycle distribution after OXA exposure is altered by PKM2 knockdown in HT29 but not HCT116 cells

**Fig 4:**
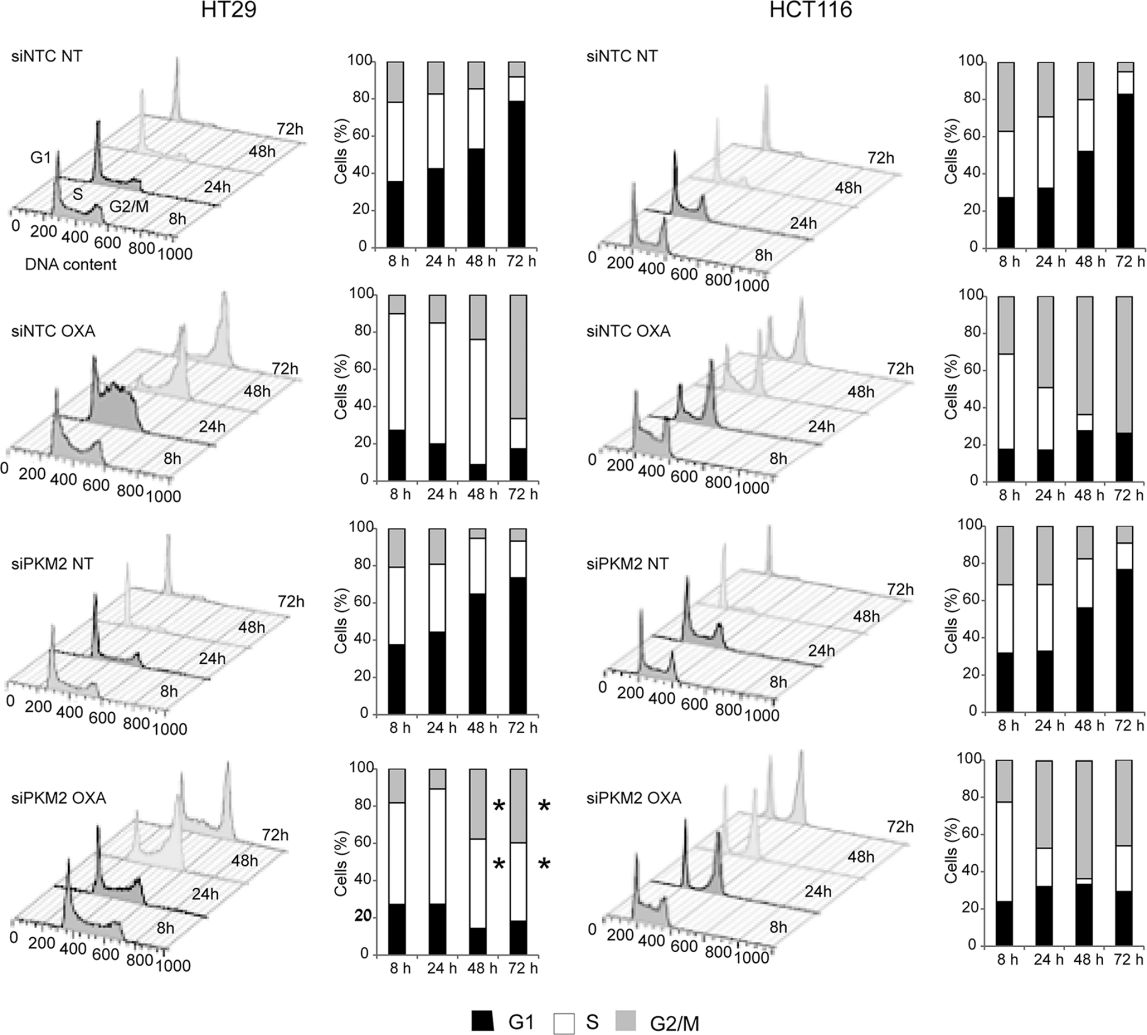
Cell cycle distribution in HT29 and HCT116 cells after PKM2 gene silencing and treatment with OXA. Both cell lines were transfected and/or exposed to 10 μM OXA for 8, 24, 48 and 72 h. After propidium iodide staining, the proportion of cells in cell cycle phases G1, S and G2/M was measured by flow cytometry and quantified by Flowjo v9.2 software. Results are representative of at least three independent experiments. *P-values* ≤ 0.05 are represented as a *.

OXA has been reported to modify protein expression and stability of p53 leading to an arrest of cells in G1 and/or G2/M principally depending on its mutational status [5, 27–29]. In order to assess if PKM2 expression, OXA and p53 mutational status led to differences in cell cycle progression, we studied cell cycle distribution throughout 72 h in PKM2 silenced and control HT29 and HCT116 cells following continuous treatment with 10 μM OXA. As it is shown in Fig 4, untreated cells displayed the same cell cycle distribution, in the presence or absence of PKM2, meaning that this protein has no effect on cell cycle regulation *per se*. However, OXA induced different responses in cell cycle control between the two cell lines. HCT116 cells treated with OXA were mainly retained in G1 and G2/M phases throughout 72 h of continuous exposure to the platinum drug and PKM2 ablation did not have a significant effect on cell cycle distribution. In contrast, treatment in HT29 cells, led them to be retained in S and G2/M phases principally. These cells were significantly affected by PKM2 knockdown in the last 48 and 72 hours of exposure (S phase cells: 67.2% siNTC vs 48% siPKM2; p = 0.05; G2/M phase cells: 66.5% siNTC vs 39.7% siPKM2; p = 0.05). siPKM2-HT29 cells did not hold more than 24 hours in any phase of the cell cycle, thus avoiding cell cycle checkpoints. These results confirm that CRC cells respond to OXA altering their cell cycle depending on p53 mutational status and PKM2 expression.

### PKM2 translocates to the nucleus in response to OXA in sensitive but not in resistant cells

**Fig 5:**
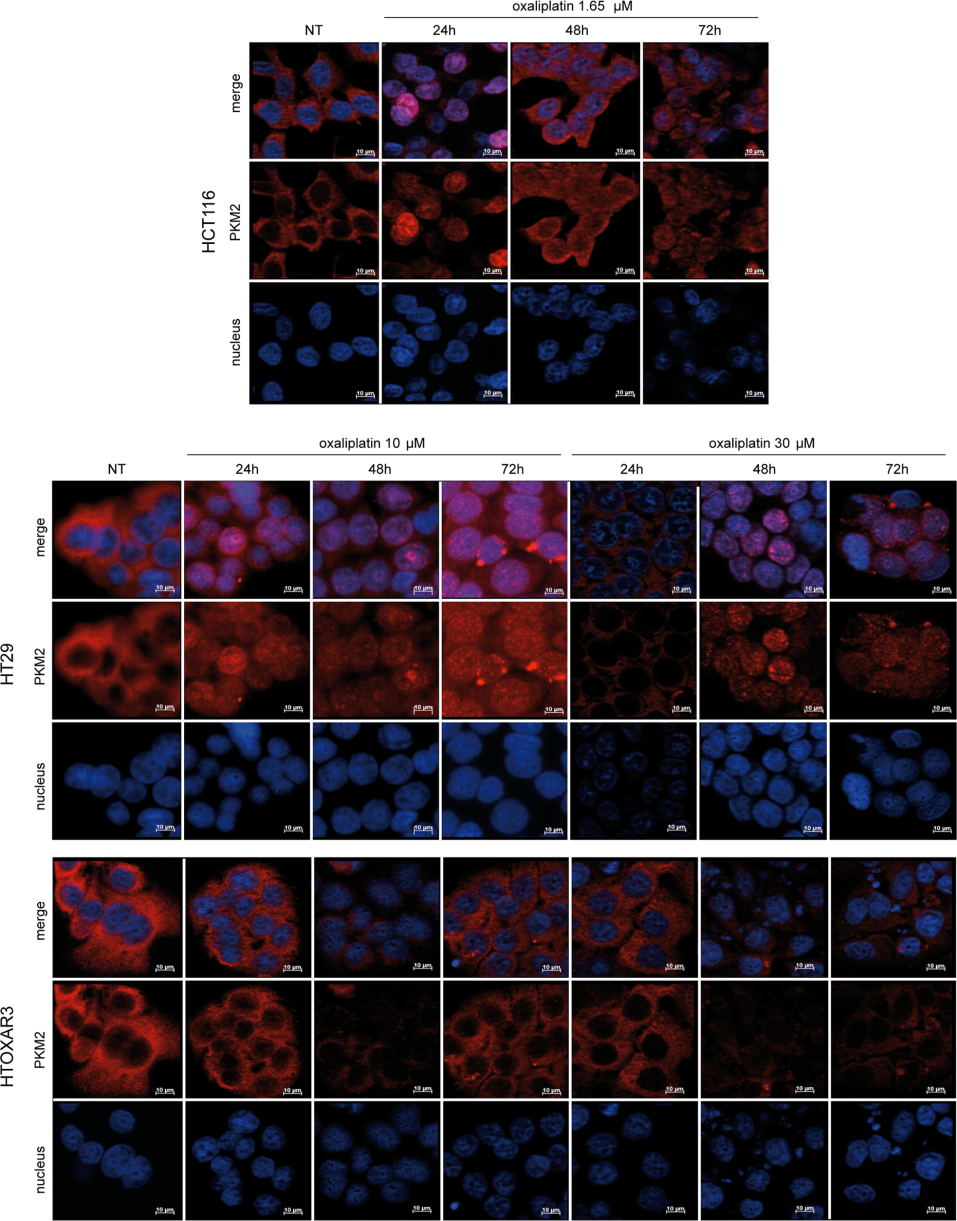
PKM2 subcellular localization in response to OXA in colorectal cancer cell lines. Immunoflourescence staining of PKM2 (red) demonstrates nuclear accumulation in HCT116 and HT29 cells after treatment with OXA in a time and dose-dependent manner but not in resistant HTOXAR3 cells. Nuclei were stained in blue. NT: Non-treated cells. Objective lens: 40x immersion oil. Scale bar: 10 μm.

It has been demonstrated that genotoxic damage caused by UV and oxidative stress stimulates PKM2 nuclear translocation to promote cell death [23]. Due to the pharmacological characteristics and mechanism of action of OXA, we wanted to know if it could induce similar changes in PKM2 subcellular localization. We treated HCT116, HT29 and HTOXAR3 cells with different doses of the drug (1.65, 10 and 30 μM) throughout 72 h and we observed PKM2 localization by using fluorescence microscopy. As it is shown in Fig 5, at basal conditions PKM2 was distributed in the cytoplasm of all the cell lines analyzed. However, exposure to OXA induced substantial changes in PKM2 localization in HCT116 and HT29 cell lines but strikingly, these changes were not observed in HTOXAR3 cells. Transient nuclear translocation of PKM2 was observed in HCT116 cells in the first 24 hours after 1.65 μM OXA exposure. Forty-eight hours later, PKM2 was relocated again in cytoplasm. At this time, most of the cells appeared shrunk and detached correlating with an increase in the amount of cell death induced by de drug. In HT29 cells, OXA induced a progressive and increasing nuclear accumulation of PKM2 along 72 h both at 10 or 30 μM. Cells with PKM2 in the nucleus showed a markedly increase in size and displayed a punctuated expression pattern of the protein. In contrast, PKM2 remained in the cytoplasm of HTOXAR3 cells through all the treatment time-points at low (10 μM) and IC_50_ (30 μM) doses of the platinum agent. Importantly, HTOXAR3 cells showed less PKM2 staining than HT29 cells corroborating our previous results [8]. HT29 siPKM2 cells were used as a control of antibody staining specificity (S2 Fig).

It has been reported that nuclear PKM2 and levels of β-catenin phosphorylation correlate with grades of glioma malignancy and prognosis [30]. Other authors have also reported basal PKM2 nuclear localization in human cell lines from different origin, including colorectal cancer [31]**.** To confirm PKM2 subcellular localization in human colorectal tumors and its possible relationship with total β-catenin, we analyzed their respective immunohystochemical staining in a tissue microarray of 41 tumor samples from metastatic CRC patients that was previously used by us [8]. Our results clearly confirmed PKM2 cytoplasmic stain in all the tumors analyzed (S3 Fig). It is noteworthy that these observations were carried out with the use of 2 different antibodies (see S1 File). PKM2 and β-catenin were highly expressed in most of the tumors analyzed but we could not find any positive correlation between PKM2 and β-catenin nuclear localization.

### PKM2 gene silencing alters the expression patterns of cell death genes in CRC cell lines in response to OXA

**Fig 6:**
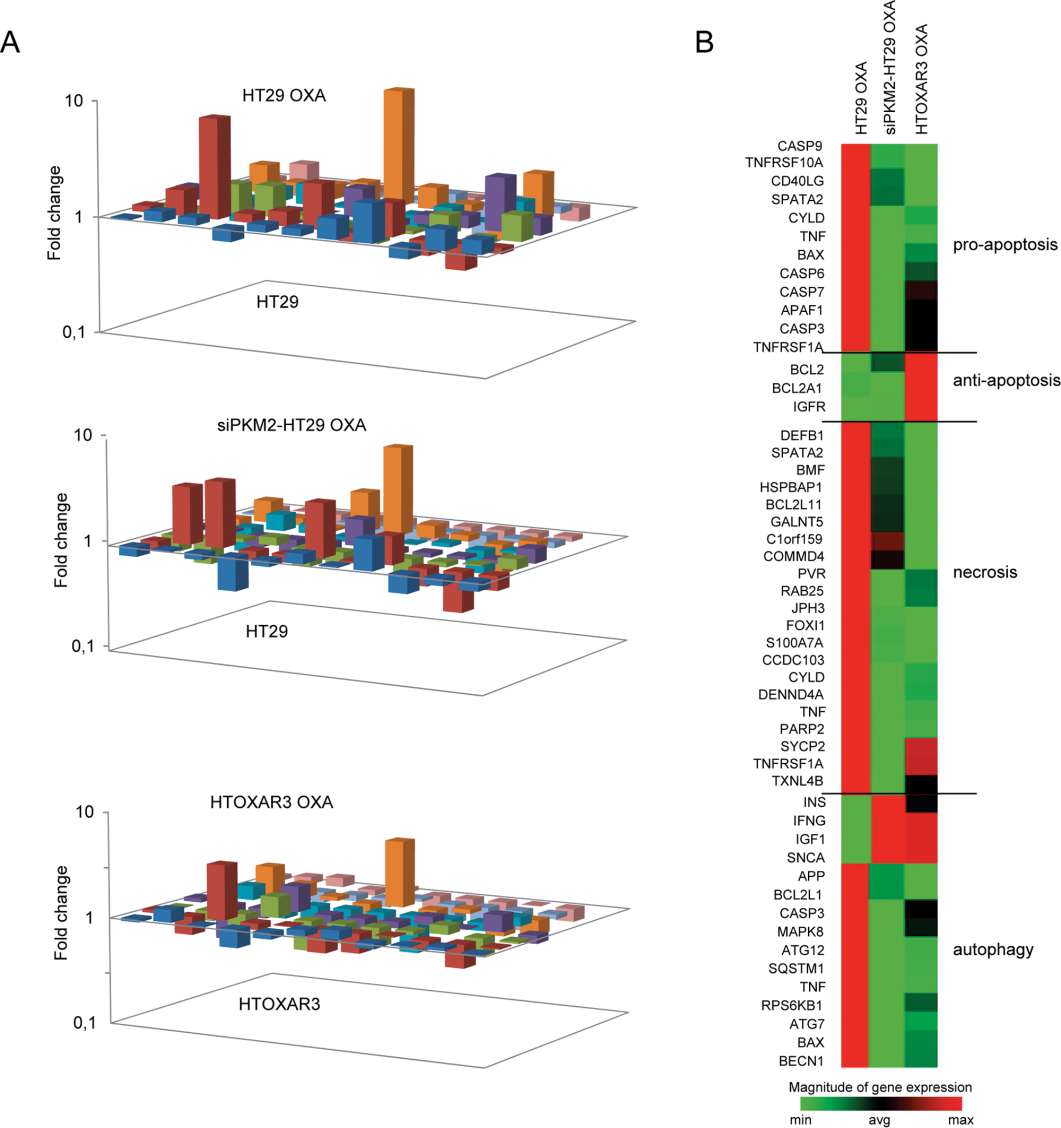
Changes in cell death genes expression patterns after PKM2 gene silencing and/or OXA treatment. A. 3-D plot showing fold changes in expression patterns after treatment with 10 μM OXA in HT29, siPKM2-HT29 and HTOXAR3 cells. B. Heat map showing up- and down-regulated genes after OXA treatment according to three different cell death pathways.

As it has been shown in this work, PKM2 translocates to the nucleus of HT29 cells in response to this platinum agent while in its OXA-resistant derivative cells it does not. Taking into account previous results associating nuclear translocation of PKM2 and the activation of caspase-independent cell death pathways [23], we speculated that PKM2 promotes transcription of genes involved in response to OXA. By using a RT^2^ profiler qPCR Array we analyzed the expression of 84 key genes related to different cell death mechanisms (apoptosis, necrosis and autophagy) in order to clarify which cell death pathway/s or gene/s play a major role in response to OXA and whether the PKM2 gene silencing affects the associated transcription patterns. To do that, HT29 cells were transfected with specific siRNAs as described before, and were treated with OXA 10 μM for 48 h in order to assure high levels of nuclear PKM2 (Fig 5). As it is shown in Fig 6A, patterns of gene expression in response to OXA were quite different among HT29, siPKM2-HT29 and HTOXAR3 cells. As we expected, the proportion of deregulated genes as a consequence of OXA exposure was considerably higher in HT29 cells as compared to HTOXAR3 since 10 μM OXA corresponds to the IC_50_ of the sensitive cells and approximately to IC_25_ for HTOXAR3 cells. Interestingly, siPKM2 cells exhibited an “intermediate” pattern of cell death genes expression. As it is clearly shown in the heat map in Fig 6B, after OXA treatment, gene expression profile of siPKM2 cells was closer to that of HTOXAR3 cells than to that of the sensitive cells. Twenty-eight of these genes showed the highest (fold-change) and/or statistically significant differences among the experimental conditions analyzed (Table 1 and S1 Table). Then, we established a rationale in order to select the best candidates to be validated specifically so that only those genes showing a statistically significant associated p-value in at least one of the experimental conditions were considered (N = 12). Among them, we chose those genes that were clearly altered in response to OXA in HT29 siNCT cells (down- or up-regulated), while they remained unaltered or changed the opposite way (up- or down-regulated) in HT29 siPKM2 and in HTOXAR3 cells under the same conditions. Bcl-2 modifying factor (BMF), met this criteria, becoming a candidate gene to study in depth.

**Table 1:** Most relevant changes in cell death genes expression patterns after treatment with OXA and/or PKM2 gene silencing.

		Comparison A				Comparison B	
Biological function	Gene	OXA	siPKM2	siPKM2 OXA	HTOXAR3	siPKM2-HT29 OXA	HTOXAR3 OXA
**Pro-apoptosis**	***BAX***	0.93	0.75	***0*.*58***	0.82	0.62	0.71
	***CASP1***	1.30	1.01	1.42	0.83	1.09	**0.45**
	***CASP3***	0.88	***0*.*60***	***0*.*56***	0.90	0.63	0.82
	***CASP7***	***0*.*76***	0.73	***0*.*48***	0.98	***0*.*62***	0.85
	***CD40***	0.76	0.72	0.76	0.97	1.00	**2.23**
	***FASLG***	**2.46**	1.52	**3.24**	1.80	1.32	**0.45**
	***TNF***	**2.86**	0.66	0.92	0.71	**0.32**	**0.36**
**Anti-apoptosis**	***BCL2***	0.85	1.88	1.06	**3.11**	1.24	1.72
	***BIRC3***	**7.28**	0.64	**4.18**	1.19	0.57	0.53
	***TNFRSF11B***	**2.44**	**2.61**	1.15	**3.66**	**0.47**	1.09
	***TRAF2***	1.32	***0*.*75***	0.83	1.26	0.63	0.94
**Apoptosis & Necrosis**	***ATP6V1G2***	1.66	**3.45**	**3.50**	**3.74**	**2.11**	1.52
	***BMF***	1.78	1.26	1.08	0.87	0.60	***0*.*37***
	***SYCP2***	0.60	0.89	**0.43**	0.96	0.70	0.96
**Necrosis**	***CCDC103***	***1*.*84***	0.91	1.19	0.81	0.65	0.69
	***COMMD4***	1.00	0.80	0.90	***0*.*88***	0.90	***0*.*78***
	***DEFB1***	1.09	0.93	0.68	1.01	0.63	**0.48**
	***DENND4A***	1.00	0.77	***0*.*69***	0.96	0.69	0.71
	***DPYSL4***	1.49	0.66	**2.38**	1.59	1.60	0.55
	***GALNT5***	**10.29**	0.60	**6.82**	1.04	0.66	**0.43**
	***MAG***	**2.16**	1.45	1.96	**2.68**	0.91	1.11
	***OR10J3***	**0.48**	0.59	0.54	0.82	1.14	1.13
	***PVR***	1.46	***0*.*52***	0.91	0.87	0.62	0.72
**Autophagy**	***ATG7***	***1*.*22***	0.80	0.95	0.76	0.78	0.82
	***CTSS***	1.16	0.92	0.88	0.52	0.76	**0.39**
	***MAP1LC3A***	1.93	0.83	1.85	0.69	0.95	**0.37**
	***MAPK8***	0.93	***0*.*58***	0.70	0.87	0.75	0.86
	***RPS6KB1***	0.99	0.63	***0*.*61***	0.88	***0*.*62***	0.74

Changes in cell death genes expression after OXA administration, PKM2 silencing and/or resistance acquisition. Legend: Comparison A: experimental condition vs. HT29 non-treated cells. Comparison B: experimental condition vs. HT29 OXA-treated cells. In bold: Values with highest Fold change value (Fold ≤ 0.5 or ≥ 2) and/or *P-value ≤ 0*.*05 (italics)*. Fold < 1 represents down-regulated genes and Fold >1 represents up-regulated genes.

**Fig 7:**
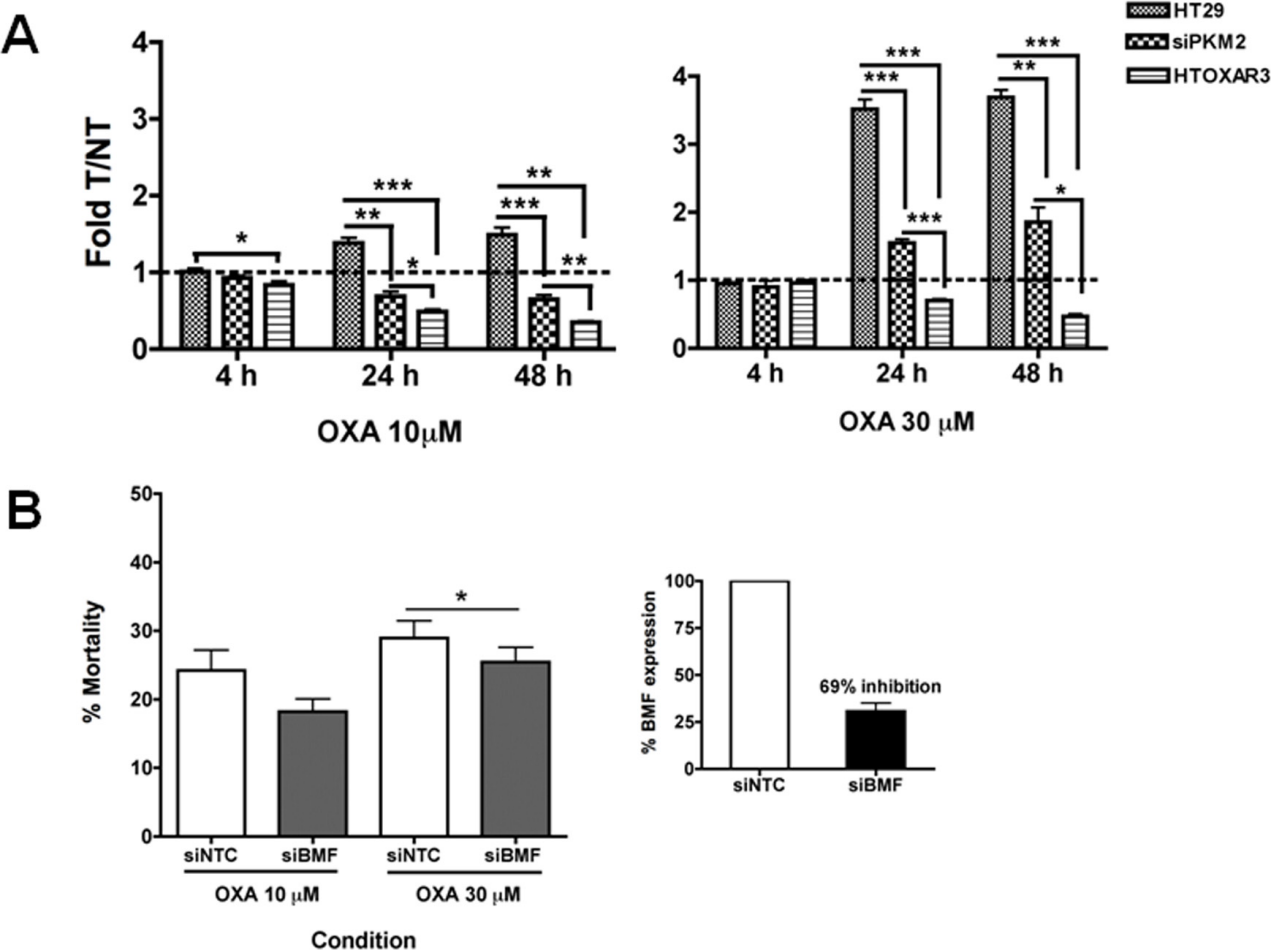
BMF is involved in oxaliplatin response and cell death at the transcriptional level A. Changes in BMF gene expression between OXA treated (T) and non-treated (NT) HT29, siPKM2- HT29 and HTOXAR3 cells. Vertical bars in the graphics represent means obtained from at least 3 independent experiments ± SD. B. Percentage of dead cells after treatment with oxaliplatin and/or BMF gene knockdown. Bars represent means obtained from at least 3 independent experiments ± SD. The Little graph shows percentage (mean ± SD) of BMF expression inhibition after siRNA transfections. * *P- value* < 0.05. ** *P-value <* 0.01; *** *P-value <* 0.001

In order to demonstrate that BMF gene expression levels were altered after oxaliplatin treatment and that this alteration in turn depends on PKM2 expression, we treated HT29-siNCT and-siPKM2 cells with oxaliplatin and compared BMF expression levels to those of HTOXAR3 cells treated under same conditions by qPCR using specific primers and Taqman probes (see material and methods section). As it is demonstrated in Fig 7A, alterations in BMF expression as a consequence of OXA administration among HT29, HTOXAR3 and siPKM2-HT29 cells were statistically significant after 24 and 48 h. While after treatment with OXA at 10 μM HT29 cells up-regulated almost 2 fold BMF gene expression, HTOXAR3 and siPKM2 cells down-regulated it. Strikingly, at a higher dose near HTOXAR3 IC_50_, HT29 cells up-regulated BMF expression almost 4-fold while resistant cells still down-regulated it. Under these conditions, siPKM2 cells showed a response similar to that of HT29 cells treated at 10 μM, elevating BMF expression up to 2 fold after 48 h. Interestingly, the expression of BMF was unchanged after oxaliplatin treatment for 24 h in both HCT116 p53 wt and null cell lines (S4 Fig). This implies a lack of involvement of BMF in oxaliplatin response and is in line with the idea that PKM2 is activating or repressing other factors in these cells. In order to assess the impact of BMF up-regulation on oxaliplatin-induced cell death, we silenced BMF expression in HT29 cells with specific siRNAs and analyzed cell death as the percentage of PI-stained cells after treatment with oxaliplatin. BMF expression was inhibited about 69% and this led to a small but statistically significant decrease in cell death after treatment with a high dose of oxaliplatin (Fig 7B). This suggests that BMF is participating in oxaliplatin-induced cell death in HT29 cells, however it probably is not the most relevant factor, which would be in line with results from the qPCR array where not only the expression of BMF was affected after oxaliplatin treatment and/or PKM2 silencing. We suggest a PKM2-mediated role of BMF (among others) in activating cell death (different from apoptosis) in response to OXA in HT29 cells that has been impaired in HTOXAR3 cells as a consequence of OXA resistance acquisition.

## Discussion

OXA-based combinations are still essential in the clinical management of advanced CRC patients. However, chemotherapy resistance remains one of the principal problems of treatment success. In a previous work, we demonstrated that PKM2 expression was diminished as a consequence of a sustained exposure to OXA in an *in vitro* model [8]. The results presented here reveal that PKM2 is involved in the response and resistance acquisition to OXA in HT29 cells through its nuclear translocation ability and by affecting expression patterns of cell death-related genes, such as BMF, which has been linked to both apoptotic and non-apoptotic cell death execution.

In order to emulate the low levels of PKM2 found in OXA resistant HTOXAR3 cells [8], we silenced PKM2 gene expression with the use of specific siRNAs in the parental cell line HT29 and assessed the effect on OXA sensitivity. After exposure to OXA, PKM2 silencing resulted as expected, in an increased resistance to the platinum drug in HT29 cells as well as in SW480 cells while strikingly, in HCT116, a p53 wild type cell line, PKM2 silencing significantly increased sensitivity to OXA, thus suggesting a possible connection among PKM2, mutational status of p53 and response to OXA. Experiments using HCT116 p53 null isogenic cells and siRNA-based inhibition of p53 in HT29 cells, showed that the observed different behavior of PKM2 in p53 wt and mutated cell lines was not dependent on p53 *per se*. A limitation of these experiments is the fact that we did not use an HCT116 p53 null cell line stably transfected with a construct encoding a p53 mutant protein (in this case the R273H mutation, the same that HT29 and also SW480 cell lines harbor). This approach would have confirmed or ruled out the role of a GOF mutation in p53 in PKM2-mediated resistance to OXA. Nevertheless, our results indicate a context-dependent behavior for PKM2 in response to OXA and lead us to speculate about the possible reasons. It is noteworthy that while HT29 and SW480 cells arise from microsatellite stable (MSS) tumors, HCT116 cells exhibit microsatellite instability (MSI) due to biallelic deletion of *MLH1* gene. It is well known that sporadic colorectal tumors evolving from the chromosomal instability pathway (CIN) are molecularly and clinically different from those coming from the microsatellite instability (MSI) pathway. The cancer genome atlas network (TCGA) reported a comprehensive and extensive molecular characterization of human colon and rectal cancer in which such differences were not only confirmed but also reinforced. They found that almost all tumors with an hypermutated genome were MSI-high, were significantly less frequently mutated in *TP53* or *APC* genes but more frequently mutated in *TGFBR2* and presented alterations in specific gene networks different from those associated with non-hypermutated MSS tumors indicating that they progress through different sequences of genetic events [32]. In such scenario it is reasonable to think that PKM2 could have different partners according to the genetic context in which it is found and consequently, behave in a different way.[11, 33][34].

We found that the increased proliferation observed in siPKM2-HT29 cells after OXA treatment was not accompanied by a decrease in apoptosis; however, a sustained S phase delay was observed in these cells as compared to siNTC-HT29, in which this delay was transient. This effect has been related to oxaliplatin resistance in p53-mutated cells [5, 6, 29]. In contrast, HCT116 cells displayed the typical retention in G1 and G2/M after treatment with oxaliplatin and no differences were observed between control and siPKM2 cells.

PKM2 translocates to the nucleus in response to oxidative stress generated by H_2_O_2_ or to genomic damage caused by UV [23]. Accordingly, we here demonstrate that oxaliplatin induced PKM2 nuclear translocation in both HCT116 and in HT29 cells but surprisingly, this movement was not observed in HTOXAR3 cells. It has been proposed that nuclear translocation of PKM2 happens after sumoylation by the SUMO-E3 ligase protein PIAS3 (inhibitor of activated STAT3) [19]. Continuous exposure of HT29 cells to oxaliplatin until obtaining of HTOXAR3 resistant cells could have modified the PKM2 protein in those residues that are important for nuclear translocation in response to oxaliplatin. For example, Anastasiou et al. showed that the exposure to acute concentrations of ROS caused oxidation of residue Cys358 [35]. Whether these or other possible modifications are behind the alteration in PKM2 ability to translocate in response to oxaliplatin in HTOXAR3 cells remains to be demonstrated.

Stetak et al. proposed a caspase-independent cell death mechanism for nuclear PKM2 under oxidative stress or DNA damage conditions [23]. In fact, it has been demonstrated that oxaliplatin induces necrotic cell death in p53-mutated cells, including HT29 [5]. Taking into account that our Annexin V/PI experiments did not show a role of PKM2 in promoting apoptosis after OXA treatment, we wanted to know if an alternative cell death pathway was activated after PKM2 translocation in response to OXA. After OXA treatment, caspases 3 and 7 were down-regulated while an up-regulation of necroptotic and autophagic genes such as *BIRC3* [36], *PVR*, *MAG*, *GALNT5* [37] and *ATG7* [35] was observed, suggesting that in our experimental conditions, cell death activated by OXA could be executed by mechanisms such as autophagy, necroptosis, or a combination of both. Moreover, PKM2 gene silencing altered the expression patterns of cell death related genes in response to OXA. One of them was the Bcl-2 modifying factor (BMF), which has been implicated not only in apoptosis and anoikis [38] but also in necroptosis execution [37, 39], a caspase-independent programmed cell death mechanism stimulated by ROS [40] and prevalently activated in p53 and apoptosis deficient cells [5, 37, 41], as well as in autophagy [42]. We found that the expression of BMF at the RNA level was deeply affected by both PKM2 knock down and OXA resistance acquisition. While in HT29 cells OXA treatment led to an increase of BMF expression, in siPKM2 and HTOXAR3 cells this increase was not observed. These differences were more evident at 30 μM, which is the IC_50_ for the resistant cells. Noteworthy, BMF gene expression remained unchanged in HCT116 p53 wt and null cells after a 24 h treatment with OXA suggesting a lack of involvement of BMF in the response to OXA in this cell line. Finally, BMF gene silencing in HT29 cells was associated with a decrease in cell death after OXA exposure. These results suggest a role of BMF on OXA-induced cell death in HT29 cells. Further studies are necessary in order to elucidate the exact mechanism by which BMF promotes cell death in response to OXA. Nevertheless, it seems that nuclear PKM2 can be directly or indirectly promoting BMF transcription in response to OXA and that resistant cells are unable to respond due to PKM2 inability to translocate to the nucleus. The way PKM2 performs this activation in transcription needs to be further explored. Gao X et al. reported a lack of known DNA-binding domain/motif in PKM2 and otherwise showed a role of PKM2 in MEK5 transcription through interaction and activation of STAT3 transcription factor [31]. It has also been shown that PKM2 directly binds to histone H3 and phosphorylates histone H3 at T11 upon EGF receptor activation. This phosphorylation is required for the dissociation of HDAC3 from the CCND1 and MYC promoter regions [43]. These are possible ways by which PKM2 could activate BMF expression in response to OXA since the latter has been reported to be controlled by HDAC8 and STAT3 [44] or by HDAC1 at the promoter level [42].

In conclusion, we here describe new evidence about non-glycolytic functions of PKM2 related to its nuclear translocation, which supports previous data about a role in activating cell death after oxidative stress and DNA damage. Specifically, this is the first time that it is described that an anticancer platinum drug induces PKM2 nuclear translocation, which is probably related to transcriptional activation of BMF, a cell death-related gene. Further experiments are guaranteed in order to uncover new roles of this moonlighting enzyme.

## Supporting Information

S1 Fig PKM2 gene silencing in human CRC cell lines HT29 and HCT116. GAPDH and PKM2 mRNA (A) and protein (B) levels 48 h post-PKM2 gene silencing by using siRNAs. C. PKM2 protein expression in HT29 and HCT116 cell lines from 24 to 96 hours post-transfection. Pictures represent one of at least three independent experiments for each condition. Vertical bars in the graphics represent ± SD.
(TIFF)

S2 Fig PKM2 inmunofluorescence staining after gene knockdown in HT29 cells. HT29 Cells transfected with NTC or PKM2 siRNAs were submitted to inmunocytochemistry staining with a specific antibody against PKM2 (red). Nuclei were stained with DAPI (blue).
(TIFF)

S3 Fig PKM2 and β-catenin expression and localization in human colorectal tumor tissues. A. Immunohistochemical staining of PKM2 and β-catenin showed PKM2 to localize in the cytoplasm. Nuclear or membrane β-catenin staining indicated the presence or not of an alteration in the Wnt pathway, respectively. B. Table showing association between PKM2 and β-catenin staining. Absolute values indicate the number of cases according with PKM2 and β-catenin stainings. Original magnification x40.
(TIFF)

S4 Fig Changes in BMF expression levels after treatment with oxaliplatin in HCT116 p53 wt and p53 null cell lines Changes in BMF gene expression between OXA treated (T) and non-treated (NT) HCT116 p53 wt and HCT116 p53 null cell lines. Vertical bars in the graphics represent means obtained from at least 3 independent experiments ± SD
(TIFF)

S1 Table qPCR array raw data. (XLSX)

S1 File Supplementary file. (DOCX)

## References

[1] Cunningham D, Atkin W, Lenz HJ, Lynch HT, Minsky B, Nordlinger B, et al Colorectal cancer. Lancet. 2010;375: 1030–47. 10.1016/S0140-6736(10)60353-4 20304247

[2] Weitz J, Koch M, Debus J, Hohler T, Galle PR, Buchler MW. Colorectal cancer. Lancet. 2005;365: 153–65. 15639298 10.1016/S0140-6736(05)17706-X

[3] Kelland L. The resurgence of platinum-based cancer chemotherapy. Nat Rev Cancer. 2007;7: 573–84. 17625587 10.1038/nrc2167

[4] Hata T, Yamamoto H, Ngan CY, Koi M, Takagi A, Damdinsuren B, et al Role of p21waf1/cip1 in effects of oxaliplatin in colorectal cancer cells. Mol Cancer Ther. 2005;4: 1585–94. 16227409 10.1158/1535-7163.MCT-05-0011

[5] Rakitina TV, Vasilevskaya IA, O'Dwyer PJ. Inhibition of G1/S transition potentiates oxaliplatin-induced cell death in colon cancer cell lines. Biochem Pharmacol. 2007;73: 1715–26. 17343830 10.1016/j.bcp.2007.01.037

[6] Toscano F, Parmentier B, Fajoui ZE, Estornes Y, Chayvialle JA, Saurin JC, et al p53 dependent and independent sensitivity to oxaliplatin of colon cancer cells. Biochem Pharmacol. 2007;74: 392–406. 17559811 10.1016/j.bcp.2007.05.001

[7] Bose D, Zimmerman LJ, Pierobon M, Petricoin E, Tozzi F, Parikh A, et al Chemoresistant colorectal cancer cells and cancer stem cells mediate growth and survival of bystander cells. Br J Cancer. 2011;105: 1759–67. 10.1038/bjc.2011.449 22045189 PMC3242606

[8] Martinez-Balibrea E, Plasencia C, Gines A, Martinez-Cardus A, Musulen E, Aguilera R, et al A proteomic approach links decreased pyruvate kinase M2 expression to oxaliplatin resistance in patients with colorectal cancer and in human cell lines. Mol Cancer Ther. 2009;8: 771–8. 10.1158/1535-7163.MCT-08-0882 19372549

[9] Plasencia C, Martinez-Balibrea E, Martinez-Cardus A, Quinn DI, Abad A, Neamati N. Expression analysis of genes involved in oxaliplatin response and development of oxaliplatin-resistant HT29 colon cancer cells. Int J Oncol. 2006;29: 225–35. 16773204 10.3892/ijo.29.1.225

[10] Rabik CA, Dolan ME. Molecular mechanisms of resistance and toxicity associated with platinating agents. Cancer Treat Rev. 2007;33: 9–23. 17084534 10.1016/j.ctrv.2006.09.006 PMC1855222

[11] Tamada M, Nagano O, Tateyama S, Ohmura M, Yae T, Ishimoto T, et al Modulation of glucose metabolism by CD44 contributes to antioxidant status and drug resistance in cancer cells. Cancer Res. 2012;72: 1438–48. 10.1158/0008-5472.CAN-11-3024 22293754

[12] Zhou R, Vander Heiden MG, Rudin CM. Genotoxic exposure is associated with alterations in glucose uptake and metabolism. Cancer Res. 2002;62: 3515–20. 12067998

[13] Yoo BC, Ku JL, Hong SH, Shin YK, Park SY, Kim HK, et al Decreased pyruvate kinase M2 activity linked to cisplatin resistance in human gastric carcinoma cell lines. Int J Cancer. 2004;108: 532–9. 14696117 10.1002/ijc.11604

[14] Sakai A, Otani M, Miyamoto A, Yoshida H, Furuya E, Tanigawa N. Identification of phosphorylated serine-15 and -82 residues of HSPB1 in 5-fluorouracil-resistant colorectal cancer cells by proteomics. J Proteomics. 2012;75: 806–18. 10.1016/j.jprot.2011.09.023 21989268

[15] Hitosugi T, Kang S, Vander Heiden MG, Chung TW, Elf S, Lythgoe K, et al Tyrosine phosphorylation inhibits PKM2 to promote the Warburg effect and tumor growth. Sci Signal. 2009;2: ra73. 10.1126/scisignal.2000431 PMC2812789 19920251

[16] Christofk HR, Vander Heiden MG, Harris MH, Ramanathan A, Gerszten RE, Wei R, et al The M2 splice isoform of pyruvate kinase is important for cancer metabolism and tumour growth. Nature. 2008;452: 230–3. 10.1038/nature06734 18337823

[17] Christofk HR, Vander Heiden MG, Wu N, Asara JM, Cantley LC. Pyruvate kinase M2 is a phosphotyrosine-binding protein. Nature. 2008;452: 181–6. 10.1038/nature06667 18337815

[18] Lv L, Li D, Zhao D, Lin R, Chu Y, Zhang H, et al Acetylation targets the M2 isoform of pyruvate kinase for degradation through chaperone-mediated autophagy and promotes tumor growth. Mol Cell. 2011;42: 719–30. 10.1016/j.molcel.2011.04.025 21700219 PMC4879880

[19] Spoden GA, Morandell D, Ehehalt D, Fiedler M, Jansen-Durr P, Hermann M, et al The SUMO-E3 ligase PIAS3 targets pyruvate kinase M2. J Cell Biochem. 2009;107: 293–302. 10.1002/jcb.22125 19308990

[20] Du H, Yang W, Chen L, Shi M, Seewoo V, Wang J, et al Role of autophagy in resistance to oxaliplatin in hepatocellular carcinoma cells. Oncol Rep. 2012;27: 143–50. 10.3892/or.2011.1464 21935576

[21] Hoshino A, Hirst JA, Fujii H. Regulation of cell proliferation by interleukin-3-induced nuclear translocation of pyruvate kinase. J Biol Chem. 2007;282: 17706–11. 17446165 10.1074/jbc.M700094200

[22] Lee J, Kim HK, Han YM, Kim J. Pyruvate kinase isozyme type M2 (PKM2) interacts and cooperates with Oct-4 in regulating transcription. Int J Biochem Cell Biol. 2008;40: 1043–54. 10.1016/j.biocel.2007.11.009 18191611

[23] Stetak A, Veress R, Ovadi J, Csermely P, Keri G, Ullrich A. Nuclear translocation of the tumor marker pyruvate kinase M2 induces programmed cell death. Cancer Res. 2007;67: 1602–8. 17308100 10.1158/0008-5472.CAN-06-2870

[24] Martinez-Cardus A, Martinez-Balibrea E, Bandres E, Malumbres R, Gines A, Manzano JL, et al Pharmacogenomic approach for the identification of novel determinants of acquired resistance to oxaliplatin in colorectal cancer. Mol Cancer Ther. 2009;8: 194–202. 10.1158/1535-7163.MCT-08-0659 19139129

[25] Alley MC, Scudiero DA, Monks A, Hursey ML, Czerwinski MJ, Fine DL, et al Feasibility of drug screening with panels of human tumor cell lines using a microculture tetrazolium assay. Cancer Res. 1988;48: 589–601. 3335022

[26] Bunz F, Dutriaux A, Lengauer C, Waldman T, Zhou S, Brown JP, et al Requirement for p53 and p21 to sustain G2 arrest after DNA damage. Science. 1998;282: 1497–501. 9822382 10.1126/science.282.5393.1497

[27] Arango D, Wilson AJ, Shi Q, Corner GA, Aranes MJ, Nicholas C, et al Molecular mechanisms of action and prediction of response to oxaliplatin in colorectal cancer cells. Br J Cancer. 2004;91: 1931–46. 15545975 10.1038/sj.bjc.6602215 PMC2409767

[28] Arnould S, Hennebelle I, Canal P, Bugat R, Guichard S. Cellular determinants of oxaliplatin sensitivity in colon cancer cell lines. Eur J Cancer. 2003;39: 112–9. 12504667 10.1016/s0959-8049(02)00411-2

[29] William-Faltaos S, Rouillard D, Lechat P, Bastian G. Cell cycle arrest by oxaliplatin on cancer cells. Fundam Clin Pharmacol. 2007;21: 165–72. 17391289 10.1111/j.1472-8206.2007.00462.x

[30] Yang W, Xia Y, Ji H, Zheng Y, Liang J, Huang W, et al Nuclear PKM2 regulates beta-catenin transactivation upon EGFR activation. Nature. 2011;480: 118–22. 10.1038/nature10598 22056988 PMC3235705

[31] Gao X, Wang H, Yang JJ, Liu X, Liu ZR. Pyruvate kinase M2 regulates gene transcription by acting as a protein kinase. Mol Cell. 2012;45: 598–609. 10.1016/j.molcel.2012.01.001 22306293 PMC3299833

[32] Cancer Genome Atlas N. Comprehensive molecular characterization of human colon and rectal cancer. Nature. 2012;487: 330–7. 10.1038/nature11252 22810696 PMC3401966

[33] Cheung EC, Vousden KH. The role of p53 in glucose metabolism. Curr Opin Cell Biol. 2010;22: 186–91. 10.1016/j.ceb.2009.12.006 20061129

[34] Vander Heiden MG, Locasale JW, Swanson KD, Sharfi H, Heffron GJ, Amador-Noguez D, et al Evidence for an alternative glycolytic pathway in rapidly proliferating cells. Science. 2010;329: 1492–9. 10.1126/science.1188015 20847263 PMC3030121

[35] Anastasiou D, Poulogiannis G, Asara JM, Boxer MB, Jiang JK, Shen M, et al Inhibition of pyruvate kinase M2 by reactive oxygen species contributes to cellular antioxidant responses. Science. 2011;334: 1278–83. 10.1126/science.1211485 22052977 PMC3471535

[36] Choi YE, Butterworth M, Malladi S, Duckett CS, Cohen GM, Bratton SB. The E3 ubiquitin ligase cIAP1 binds and ubiquitinates caspase-3 and -7 via unique mechanisms at distinct steps in their processing. J Biol Chem. 2009;284: 12772–82. 10.1074/jbc.M807550200 19258326 PMC2676007

[37] Hitomi J, Christofferson DE, Ng A, Yao J, Degterev A, Xavier RJ, et al Identification of a molecular signaling network that regulates a cellular necrotic cell death pathway. Cell. 2008;135: 1311–23. 10.1016/j.cell.2008.10.044 19109899 PMC2621059

[38] Elkholi R, Floros KV, Chipuk JE. The Role of BH3-Only Proteins in Tumor Cell Development, Signaling, and Treatment. Genes & cancer. 2011;2: 523–37. 21901166 10.1177/1947601911417177 PMC3161420

[39] Tischner D, Manzl C, Soratroi C, Villunger A, Krumschnabel G. Necrosis-like death can engage multiple pro-apoptotic Bcl-2 protein family members. Apoptosis. 2012;17: 1197–209. 10.1007/s10495-012-0756-8 22971741 PMC4918797

[40] Zhang DW, Shao J, Lin J, Zhang N, Lu BJ, Lin SC, et al RIP3, an energy metabolism regulator that switches TNF-induced cell death from apoptosis to necrosis. Science. 2009;325: 332–6. 10.1126/science.1172308 19498109

[41] Galluzzi L, Maiuri MC, Vitale I, Zischka H, Castedo M, Zitvogel L, et al Cell death modalities: classification and pathophysiological implications. Cell Death Differ. 2007;14: 1237–43. 17431418 10.1038/sj.cdd.4402148

[42] Contreras AU, Mebratu Y, Delgado M, Montano G, Hu CA, Ryter SW, et al Deacetylation of p53 induces autophagy by suppressing Bmf expression. The Journal of cell biology. 2013;201: 427–37. 10.1083/jcb.201205064 23629966 PMC3639396

[43] Yang W, Xia Y, Hawke D, Li X, Liang J, Xing D, et al PKM2 phosphorylates histone H3 and promotes gene transcription and tumorigenesis. Cell. 2012;150: 685–96. 10.1016/j.cell.2012.07.018 22901803 PMC3431020

[44] Kang Y, Nian H, Rajendran P, Kim E, Dashwood WM, Pinto JT, et al HDAC8 and STAT3 repress BMF gene activity in colon cancer cells. Cell death & disease. 2014;5: e1476. 25321483 10.1038/cddis.2014.422 PMC4237248

