# Supplementary figures and images for: PKM2 Subcellular Localization Is Involved in Oxaliplatin Resistance Acquisition in HT29 Human Colorectal Cancer Cell Lines
Source: PLoS One. 2015 May 8;10(5):e0123830. doi: 10.1371/journal.pone.0123830 (PMC4425499; doi:10.1371/journal.pone.0123830)

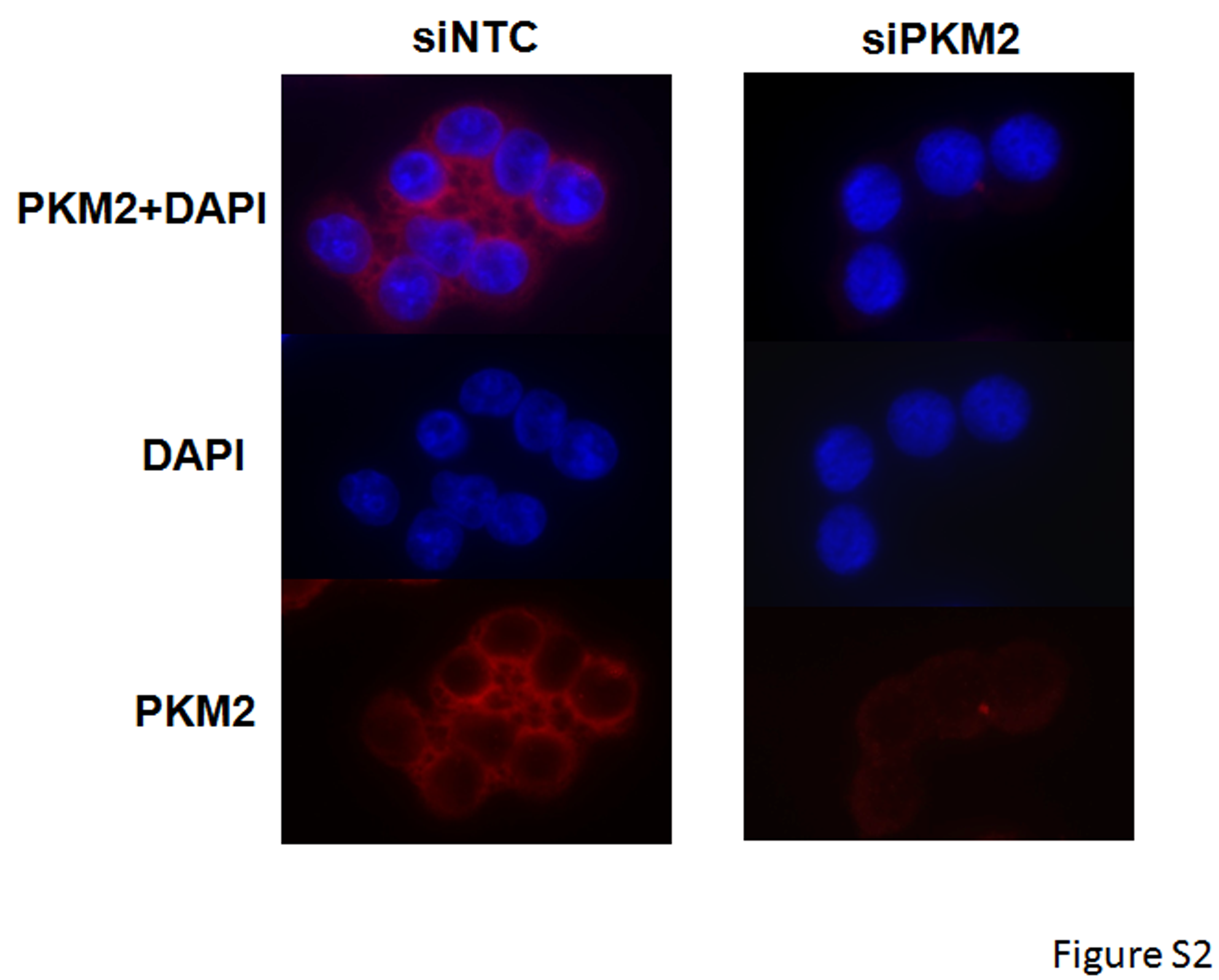

Supplement: S2 Fig — HT29 Cells transfected with NTC or PKM2 siRNAs were submitted to inmunocytochemistry staining with a specific antibody against PKM2 (red). Nuclei were stained with DAPI (blue). (TIFF) [file pone.0123830.s002.tiff]

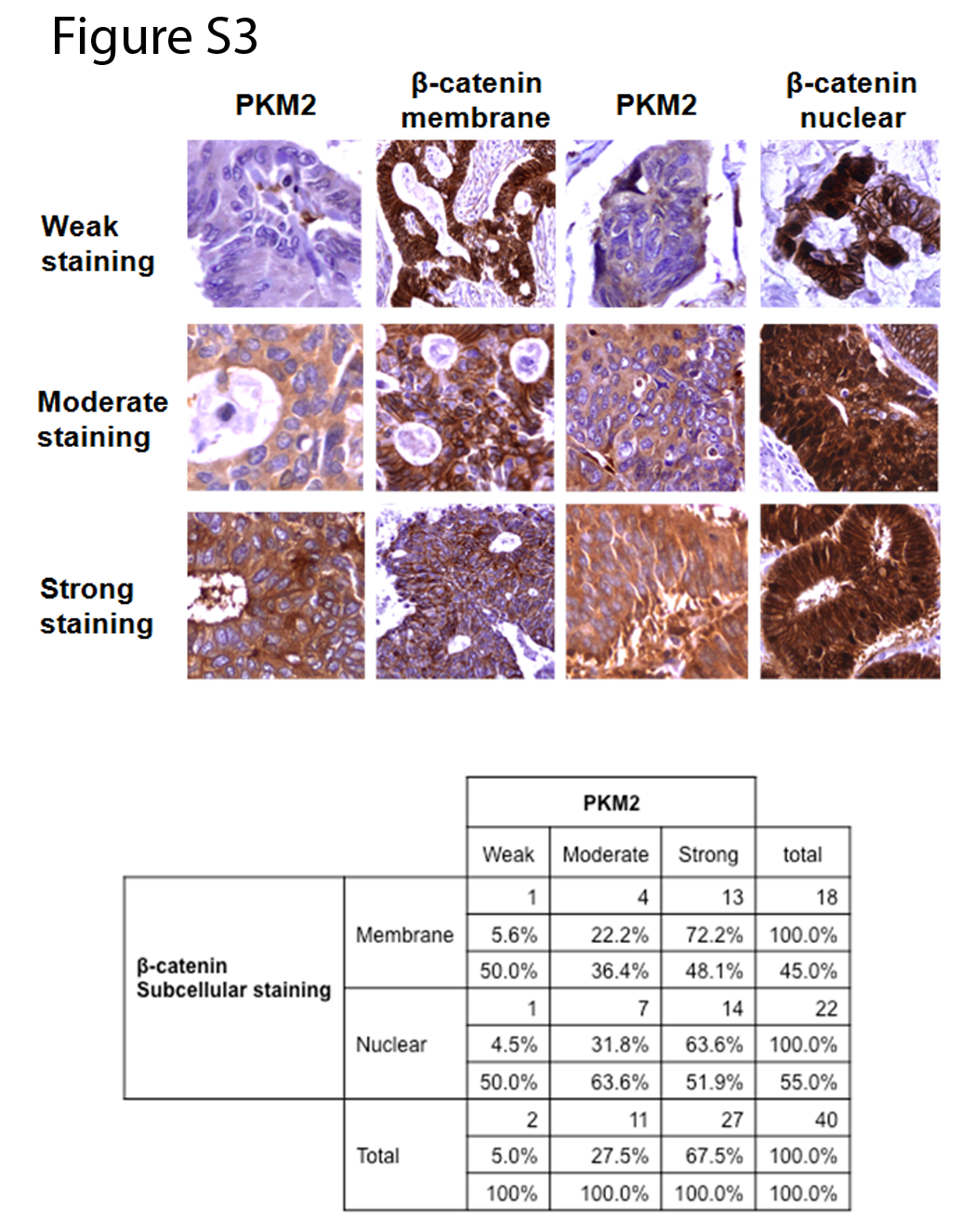

Supplement: S3 Fig — A. Immunohistochemical staining of PKM2 and β-catenin showed PKM2 to localize in the cytoplasm. Nuclear or membrane β-catenin staining indicated the presence or not of an alteration in the Wnt pathway, respectively. B. Table showing association between PKM2 and β-catenin staining. Absolute values indicate the number of cases according with PKM2 and β-catenin stainings. Original magnification x40. (TIFF) [file pone.0123830.s003.tiff]

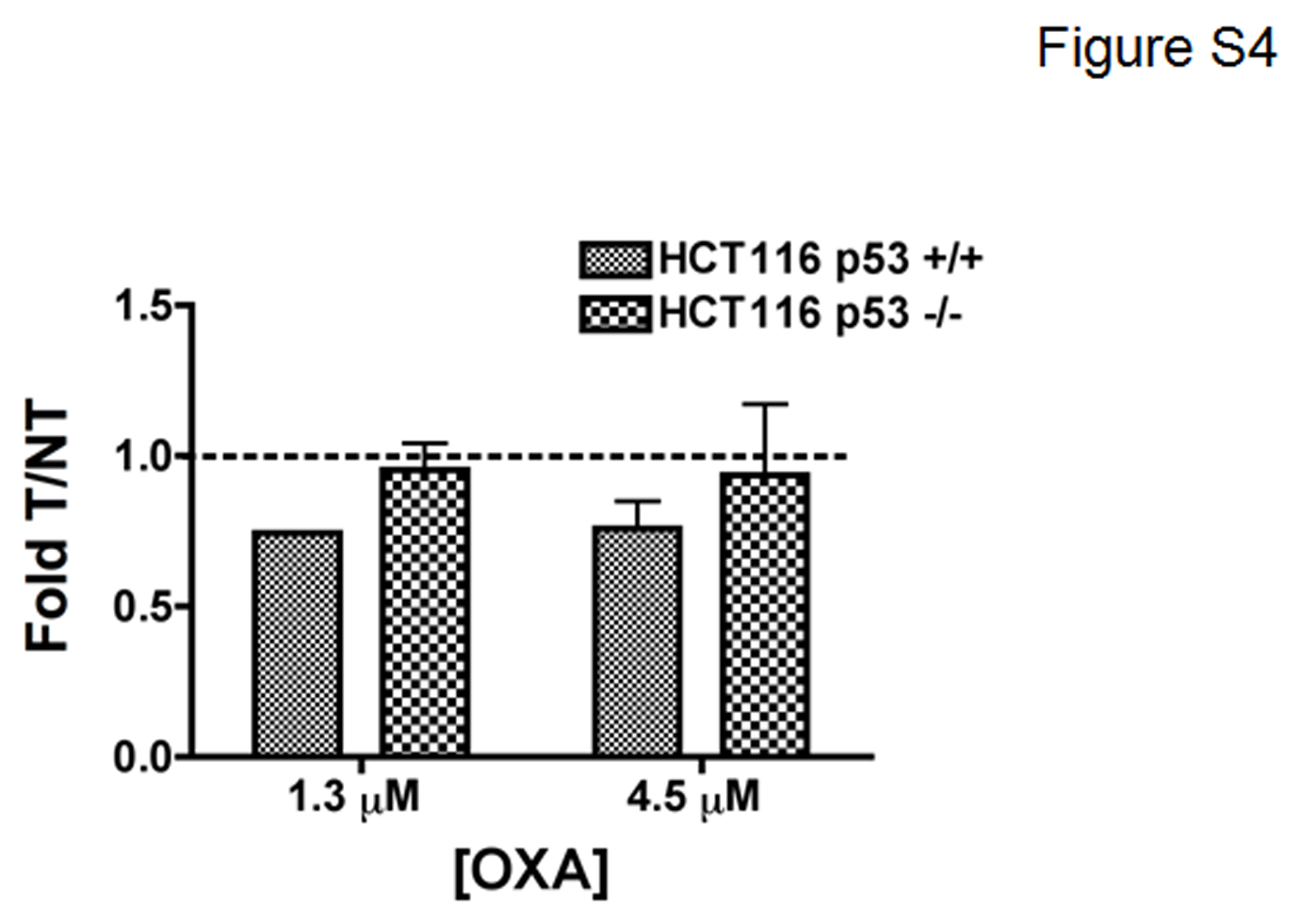

Supplement: S4 Fig — Vertical bars in the graphics represent means obtained from at least 3 independent experiments ± SD (TIFF) [file pone.0123830.s004.tiff]
