# Supplementary material for: PKM2 Subcellular Localization Is Involved in Oxaliplatin Resistance Acquisition in HT29 Human Colorectal Cancer Cell Lines
Source: PLoS One. 2015 May 8;10(5):e0123830. doi: 10.1371/journal.pone.0123830 (PMC4425499; doi:10.1371/journal.pone.0123830)
Supplement: S1 File — (DOCX) [file pone.0123830.s006.docx]

**Supplementary data.**

**Patients’ samples and Immunohistochemistry**

A tissue microarray from 41 aCRC patients was generated as described previously (8). Sections were stained using a fully automated staining system for immunohistochemistry (Benchmark, XT, Ventana Medical Systems, Inc, Tucson, Arizona, USA). The primary antibodies used were anti-PKM2 (3198S, Cell Signaling Technology, Inc) at dilution 1/20 and anti-β-catenin (SC-7963, Santa Cruz Biotechnology) at dilution1/100. Cytoplasmic immunostaining of HT29 cells was used as anti-PKM2 positive control. Membrane immunostaining in normal colonic mucosa served as anti- β-catenin positive control. A nonimmnune mouse serum was used as a negative control. PKM2 intensity staining was semiquantitative evaluated in weak, moderate and strong. Staining for β-catenin was stratified according to nuclear versus cell membrane positivity. Signed informed consent was obtained from each patient, and the Clinical Research Ethical Committee from Hospital Germans Trias I Pujol provided approval for the study.
